# Physical activity levels, agility skills and speed among female basketball players compared to non-basketball players in Saudi Arabia: A cross-sectional study

**DOI:** 10.1371/journal.pone.0312431

**Published:** 2024-11-22

**Authors:** Alaa M. Albishi

**Affiliations:** Department of Health Rehabilitation Sciences, College of Applied Medical Sciences, King Saud University, Riyadh, Saudi Arabia; Erzurum Technical University: Erzurum Teknik Universitesi, TÜRKIYE

## Abstract

**Background:**

Research findings indicate a concerning prevalence of physical inactivity among Saudi females, which has been associated with low physical performance levels. To address this challenge, it is crucial to promote sports participation, particularly in activities such as basketball. However, despite its potential benefits, the specific effects of basketball engagement among Saudi females remain largely unexplored. Therefore, this study investigated the relationship between physical performance and basketball playing among female collegiate players compared to non-basketball players in Saudi Arabia. Also, this study examined the effect of years of playing basketball on the basketball players group’s speed, agility, and physical activity levels.

**Methods:**

The participants were divided into two groups: a non-athletic control group of 20 participants (age: 22 ± 2.0 years; weight: 55.6 ± 7.7 kg; height: 157.7 ± 5.5 cm) and a basketball group of 18 participants (age: 20 ± 3.3 years; weight: 60.6 ± 12 kg; height: 163.8 ± 6.3 cm).To examine the relationship between playing basketball and physical performance levels, this study included 38 female participants divided into a non-athletic control group (N = 20) and a basketball group (N = 18). To evaluate physical performance, the 10-Meter Walk Test (10MWT), Modified Agility T-Test (MAT), and International Physical Activity Questionnaire (IPAQ) were used.

**Results:**

Basketball players demonstrated significantly superior performance in the 10-MWT (Mann-Whitney-test = 1.7, p < .05) and MAT (Mann-Whitney-test = 9.6, p < .001) compared to the non-athletic participants. Furthermore, IPAQ scores were significantly correlated with speed and agility (p < 0.05).

**Conclusion:**

Women who regularly played basketball exhibited superior physical activity levels and higher physical performance in speed and agility compared to those who were less active. However, no significant differences between the years of playing and any other variables were found. Nevertheless, these findings seek to contribute valuable insights into the potential benefits of basketball playing among Saudi women.

## 1. Introduction

The prevalence of physical inactivity among women is a growing public health problem in Saudi Arabia [1–3]. Physical inactivity and low physical performance have been associated with many health problems, such as non-communicable diseases [1–4]. A recent systematic review demonstrated high inactivity levels among the Saudi population, with a high percentage of inactive females compared to males, and indicated that the proportion of Saudis who were at risk of inactivity was exceedingly higher than those at risk for other coronary heart diseases [1]. According to the World Health Organization (WHO), physical inactivity is responsible for 6% of global mortality and is the fourth leading cause of death [5]. In addition, a prospective cohort study published in 2011,conducted in Taiwan among 416,175 individuals between the years 1996 and 2008 calculated hazard ratios for mortality risks and used the life table method to identify life expectancy associated with physical inactivity [6]. The study demonstrated that the proportion of physical inactivity was higher in women than in men. It also showed that inactive individuals had a 17% increased risk of mortality compared to individuals who exercised for 15 minutes/day. In addition, those who exercised for 15 minutes/day or an average of 92 minutes per week had a 14% reduced risk of all-cause mortality and a 3-year longer life expectancy than inactive individuals. These results were demonstrated across all age groups and both sexes, as well as among those with cardiovascular disease risks.

The WHO has recommended physical activity levels to promote health for adults aged 18–64 years is to do at least 150 minutes of moderate-intensity aerobic PA throughout the week, or at least 75 minutes of vigorous-intensity aerobic PA throughout the week, or an equivalent combination of moderate- and vigorous-intensity as a way of health enhancement [5]. Recently, studies in this area have begun to emphasize the female population, reporting an increase in physical inactivity and thus in related obesity and non-communicable diseases, especially cardiovascular disease, diabetes, and high blood pressure [7]. A study by Khalaf and colleagues [8] showed a high prevalence of female students not meeting the WHO recommendations for physical activity at a vigorous-intensity level (85%), even though evidence has shown that physical activity reduces the risk of diabetes, stroke, ischemic heart diseases, and breast and colon cancers [5]. The WHO guidelines also outline some of the longer-term benefits of physical activity, including improvements in aerobic or cardiovascular endurance, muscle strengthening, flexibility, and movement velocity [9]. For instance, improving physical activity has been associated with enhancing gait speed, agility, balance performance, and survival [10–14].

Regarding enhancing physical activity levels, a recent study shows that organized sports are more crucial for individuals’ overall physical activity than ever before, and those who are usually less physically active can be motivated through sports [15]. For instance, playing sports such as basketball has been associated with improving gait speed and agility performance [15–19]. A recent study reported that the physical characteristics of basketball players can predict their superior speed and agility abilities [20]. In addition, a study by Delextrat and colleagues [16] reported that players with better speed, strength, and agility abilities, according to playing position, had advantages in game situations [16]. Furthermore, research suggested that a significant relationship exists between jump performance, running speed, and agility in basketball players [21–23]. Thus, improving agility and speed performance is considered essential for basketball players. In fact, it is crucial to consider parameters, including physical activity level, agility, and gait speed, when measuring basketball players’ performance, as demonstrated in previous studies [15–23].

Despite the global popularity of basketball, insufficient research has been published regarding the impact of basketball on the physical performances of Saudi women concerning their physical activity levels [1–3]. Although previous studies have revealed differences in physical performance and activity levels between different populations, such as young people versus older adults and athletes versus nonathletes,[5, 8, 11, 15–25] which could result from differences in environmental factors, nutrition, variation in growth, age, sex, or sport and physical activity levels [26–29]. To our knowledge, no previous study has investigated the relationship between physical performance and basketball playing using female collegiate players compared to their low-active student peers in Saudi Arabia. Thus, this study will take the first step to compare physical performance and activity levels between basketball players and non-players women in Saudi Arabia. Additionally, we aim to examine the effect of years of playing basketball on the basketball players group’s speed, agility, and physical activity levels. Given the potential benefits of playing basketball, we hypothesized that playing basketball enhances the physical performance and activity levels of Saudi women. If playing basketball enhances the physical performance levels of Saudi women in various ways, then more work should be done to implement basketball as a sport among Saudi women. Moreover, access to sports and increased awareness should be provided to prevent the complications associated with a lack of activity among Saudi women.

## 2. Materials and methods

### 2.1. Study population and procedures

This study was conducted with a cross-sectional design. An initial power analysis (G*Power, University Duesseldorf, Duesseldorf, Germany) was performed to determine the sample size [30, 31]. Moreover, due to the lack of similar research among Saudi women athlete groups, the sample size was estimated based on previous research conducted among Saudi women horseback riders to reveal differences in walking speed between riders and non-athletes control group similar to this study age group, using alpha = 0.05, power = 0.95, d = 1.74 in the G*power program revealed a minimum of 10 in each group (total of 20) [32]. Similarly, a minimal sample of 10 per group was used to demonstrate differences in walking speed and agility among different basketball players, as reported in a previous study [16]. However, we increased our sample size to count for any withdrawal or missing data. Therefore, a total of 38 female students aged between 20 and 26 years from King Saud University were recruited. Subjects were recruited voluntarily through direct open invitations for all female students. Subjects were divided into two groups: basketball players (N = 18) and non-basketball players (N = 20). The inclusion criterion for the basketball player group was (1) play basketball at least twice a week for at least one year, similar to previous studies [15, 29]. The inclusion criterion for the non-athletic control group was (1) not engaged in at least 150 minutes of moderate-intensity physical activity per week or equivalent, as recommended by WHO [15]. The exclusion criteria were (1) previous lower extremity musculoskeletal injuries or low back pain; (2) any surgery in the lower or upper extremities; (3) participation in any additional activity or sports; (4) chronic diseases such as diabetes and hypertension; or (5) a history of cardiovascular and pulmonary disease. The study received ethical approval from the Institutional Review Board at King Saud University (no. E-23-7899) and was conducted from 20 August 2023 to 20 February 2024. Guidelines for reporting results were used, using the observational descriptive studies (STROBE Statement) S1 Checklist.

Data collection took place in a designated laboratory room at the College of Applied Sciences at King Saud University to carry out the experiment and to ensure a similar environmental setup for both groups. Also, the research procedures and objectives were explained to all participants, and their informed consent was obtained before participating in the study. Informed consent entails details about the participants’ right to withdraw from the study at any time and ensures the confidentiality of the information they are given. All participants were asked to sign a written consent form before participating in the study. To protect the participants’ privacy and avoid potential sources of bias during data analysis, each participant was assigned a unique code, and the data collected sheets were securely stored in a locked online folder to maintain the subjects’s confidentiality.

### 2.2. Outcome measures

#### 2.2.1. Physical characteristics

Data collection started with recording all participants’ physical characteristics, such as height, weight, and body mass index (BMI). Height was measured using a stadiometer to the nearest 0.1 centimeter. Weight was measured using an electronic scale to the nearest 0.1 kilogram (kg). The BMI was calculated by dividing the person’s weight in kg by the square of height in meters (m), [BMI = kg/m^2^].

#### 2.2.2. Ten-Meter Walk Test (1OMWT)

**Fig 1:**
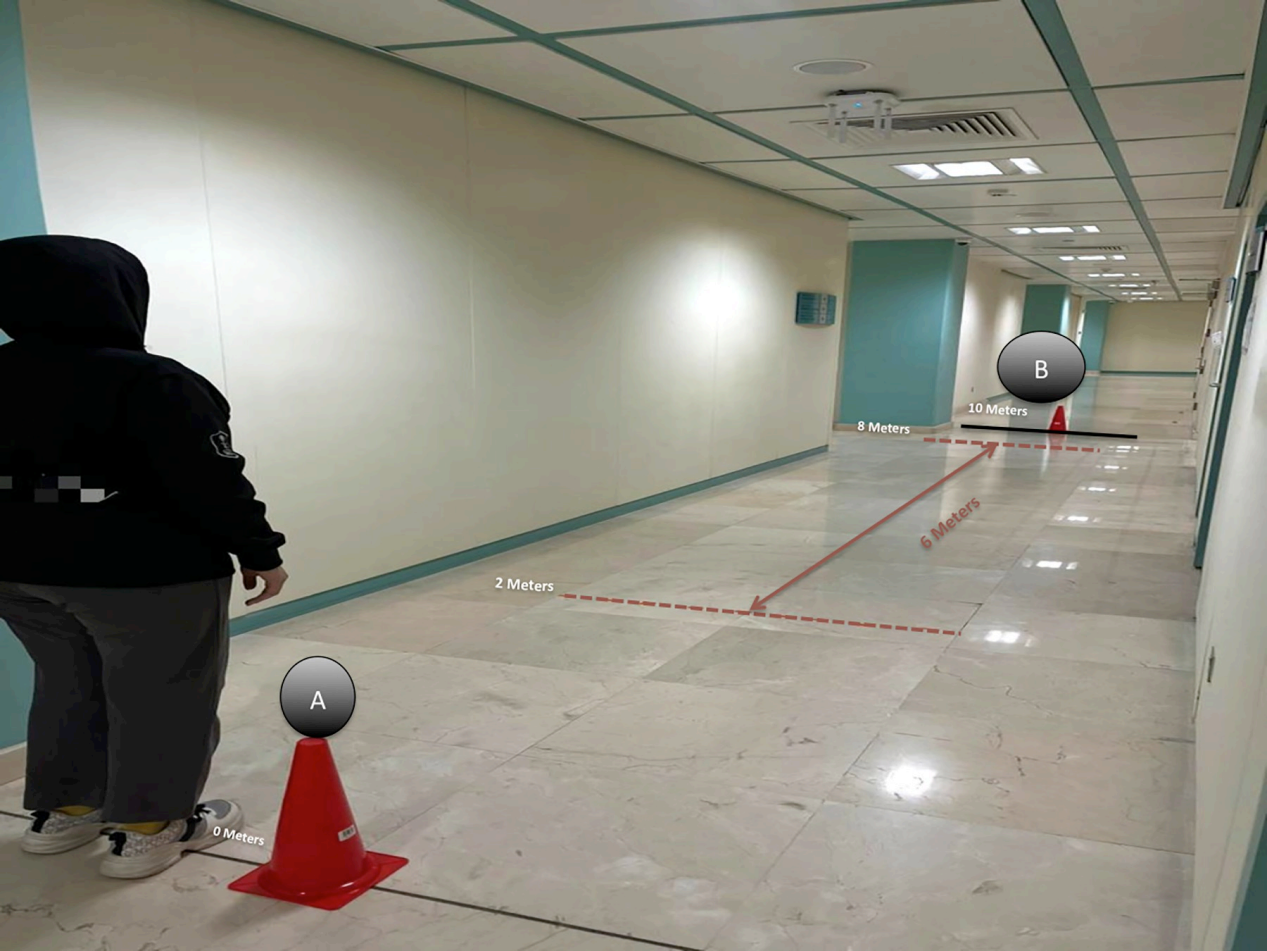
10 Meters walking test (10MWT). Walk from (A) to (B).

Gait speed was measured using the Ten-Meter Walk Test (10MWT), which assesses an individual’s comfortable and fast gait speed [33, 34]. In the 10MWT, the middle six meters were divided by the total time (in seconds) taken to ambulate and were recorded in m/s. Before the test started, a clear pathway of 10 meters (32.8 feet) in length was marked. A marker was placed at the start and end points of the 10-meter walkway; other marks were added in the middle at 6 m, 2 m, and 8 m. The time was recorded when the foot crossed the line of the 2-meter mark and stopped when the foot crossed the line of the 8-meter mark to allow the participant to accelerate and decelerate (Fig 1). The time was measured with a stopwatch. The participant started the test with a comfortable walking speed of 0 meters. The participant was instructed, “Walk at your own comfortable pace and stop when you reach the last mark.” The procedure was used for the fast walking speed assessment, but the participant was instructed, “Walk as fast as you can safely walk and stop when you reach the far mark.” Two trials were administered for the comfortable walking speed, followed by two trials for the fast walking speed, and the average was taken of the trials for the two walking speeds (m/s). The total time to administer the test was approximately five minutes. The 10MWT has excellent test-retest reliability for normal speed (ICC = .984; CI = .979-.988) and the fastest gait speed (ICC = .972 and CI = .964-.978) for a healthy adult [34].

#### 2.2.3. Modified Agility Test (MAT)

**Fig 2:**
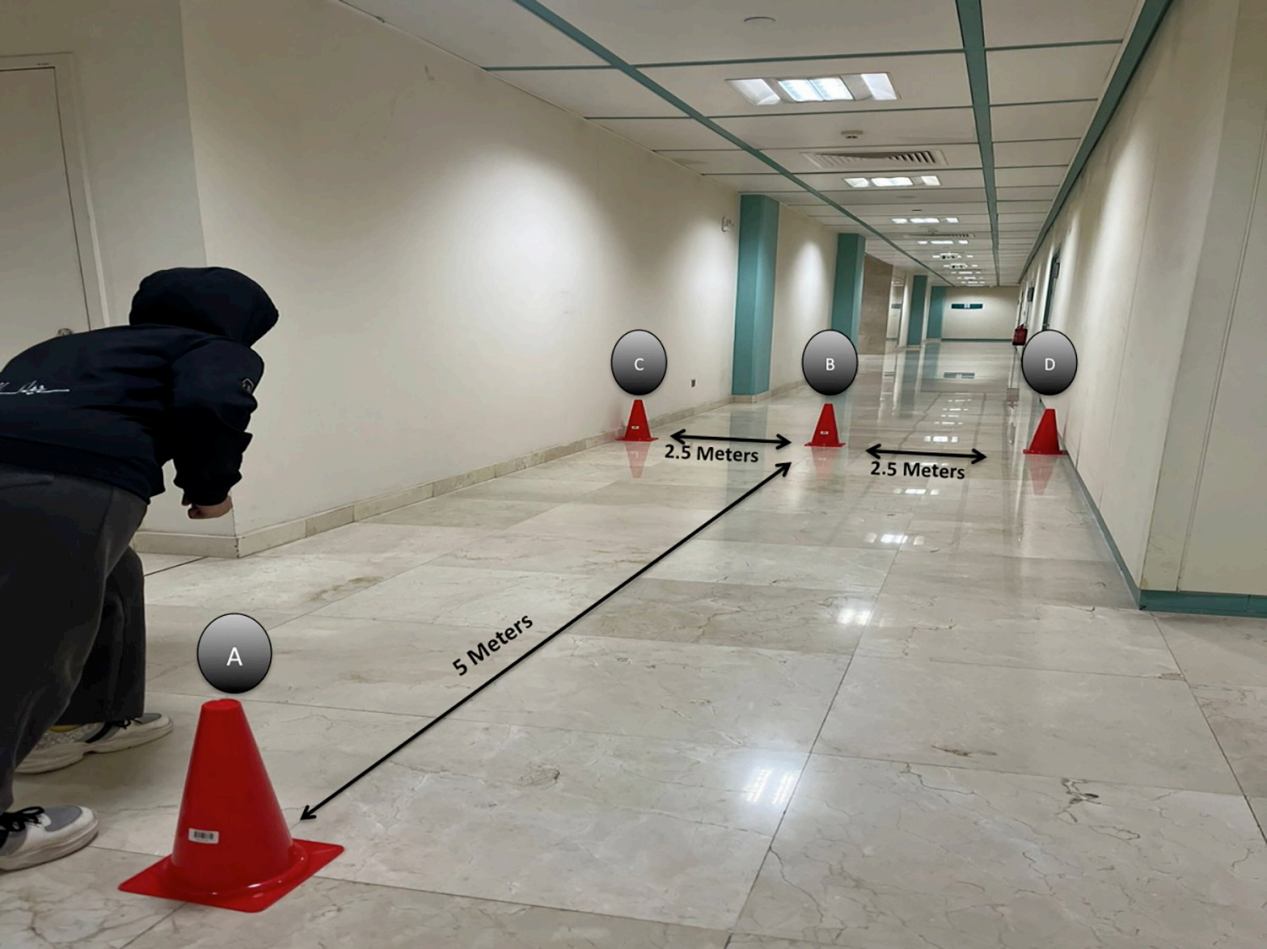
Modified Agility Test (MAT). Sprint in a forward direction from cones (A) to (B); then shuffle to left cone (C) then shuffle to the right cone (D), then shuffle back to cone (B) before running backward to the starting position cone (A).

Agility was measured using the Modified Agility T-test (MAT), which is the ideal test for athletes to evaluate agility, as it includes basic movements of forward, lateral, and backward running [35, 36]. Participants sprinted in a straight line from a standing point and squatted to touch the base of a cone or any landmark they put 5 m away with their right hand. They then shuffled sideways to their left without crossing their feet to another landmark 2.5 m away from the first and squatted to touch the base with their left hand. Next, they shuffled to the right side to a third landmark placed 5 m away and squatted to touch the base with the right hand. They finally shuffled back to the second landmark, touched the base with the left hand, and then ran back to the start line (Fig 2). The fastest of three attempts was recorded. The time to completion was measured using a digital stopwatch. This test has already shown excellent reliability (ICC = .92; 95%CI = .84-.96) [36].

#### 2.2.4. Arabic short form of the International Physical Activity Questionnaire (IPAQ)

The International Physical Activity Questionnaire (IPAQ) short-version format was used to evaluate the participant’s physical activity level [37]. It has seven items regarding information about vigorous- and moderate-intensity physical activities, time spent walking, and sedentary activity during the previous seven days. The questionnaire encompasses domains such as transport and household, fitness, and sports activities [38–41]. Physical activities (PA) were given metabolic equivalent (MET) values based on the Compendium of Physical Activity [39]. Computation of the total PA minutes per week required multiplying the duration (in minutes/day) and the frequency (days/week) for walking moderate-intensity and vigorous-intensity bouts (of at least 10 min each). Energy expenditure was calculated by multiplying the time/week for each type of activity by the MET intensity value for that activity (MET-minutes per week) for walking, moderate-intensity PA, and vigorous-intensity [37, 39]. MET is the energy cost of an activity relative to the resting metabolic rate (3.5 ml O2/kg per minute) [37]. IPAQ defines moderate physical activity as doing some activity that is more than likely equivalent to one-half of an hour of at least moderate-intensity physical activity on most days [40]. This is equivalent to 3–6 metabolic equivalents (MET) based on the Compendium of Physical Activity [39]. Vigorous physical activities are defined as doing approximately one hour of activity per day or more of at least a moderate-intensity activity level [40]. Their metabolic equivalent value is above 6 MET. Suppose the participant did not meet any of the moderate or high physical activity level criteria; in that case, they were considered to have a low level of physical activity. The participants completed the questionnaire at their homes. The IPAQ short questionnaire has been shown to be reliable and valid [37, 40]. The ICC for the Arabic version of IPAQ short form has already demonstrated moderate to good reliability (ICC ranged from 0.63 to 0.74 and was 0.79 for the total re-called weekly PA in MET*min per week IPAQ) [37].

### 2.3. Statistical analysis

Statistical analysis was performed using the Statistical Package for the Social Sciences (SPSS) version 25.0 software (SPSS Inc., Chicago, IL, USA). The data were evaluated to see whether they had normal distributions with no missing data. The Shapiro-Wilk test did not verify all the outcome measures regarding the normality assumption [42]. Descriptive statistics, such as means and standard deviations, summarized the two groups’ characteristics, including age, weight, height, BMI, and various physical performance measures.

Moreover, inferential statistics were applied to assess the significance of differences between the basketball player and non-athletic control groups. A p-value < 0.05 was considered statistically significant. The non-parametric Mann-Whitney U test was utilized for continuous variables, and the Fisher Exact test was used for categorical variables, such as IPAQ scores, to determine whether there were significant differences between the groups (Table 2).

Additionally, independent samples t-tests were used for normally distributed data. Meanwhile, the Mann-Whitney U and Spearman’s correlation rho efficient tests were used for non-normally distributed data. Confidence intervals (CI; 95%) were calculated to estimate the precision of the sample statistics and to provide insights into the range of plausible values for population parameters. Cohen’s standardized effect size (d) was used to determine the magnitude of differences, using the following thresholds: 0.0 to 0.2, trivial; 0.2 to 0.6, small; 0.6 to 1.2, moderate; 1.2 to 2.0, large; >2.0, very large [43]. Furthermore, the following scale was used to assess the magnitude of Spearman’s correlations and their differences: 0.00 to 0.10, trivial; 0.11 to 0.29, small; 0.30 to 0.49, moderate; and 0.5 to 1, large [44, 45]. In addition, Spearman’s correlation was employed for the basketball group to examine the relationships between years of playing and all independent variables.

## 3. Results

In terms of physical and sports experience characteristics (Table 1), we found that the basketball players had a significantly higher mean height (p = 0.003; d = 0.929, moderate effect size (ES)), right leg length (p = 0.008; d = 0.839, moderate ES), and left leg length (p = 0.01; d = 0.811, moderate ES) compared to the non-athletic women. Regarding specific physical performance tests, the basketball players demonstrated significant differences in the 10 MWT normal (p = 0.012; d = 0.597, small ES) and MAT (p < 0.05; d = 1.676, large ES) compared to the non-athletic control group, indicating better speed and agility as the basketball players demonstrated better speed and agility performance on average (i.e., shorter times in seconds) than the non-athletic group. However, no significant differences were found in the other measures between the two groups (Table 1).

**Table 1:** Description analysis of the study variables.

Variables	Basketball			Non-active			CI 95%				
	Mean/Median		SD/ IQR	Mean/Median		SD/ IQR	Lower	Upper	Cohen’s d		P*
**Physical characteristics**											
Age	20.00^b^	3.25^c^			22.00^b^	2.00 ^c^	-1.286	1.953	0.137		0.737^a^
Weight	60.56	12.02			55.55	7.74	-1.58	11.60	0.49		0.13
Height	163.78	6.31			157.65	5.51	2.24	10.02	0.93		0.00*
BMI	22.34	3.83			22.21	2.93	-2.13	2.40	0.04		0.90
Right leg length	89.47	4.46			84.63	5.95	1.354	8.34	0.84		0.01*
Left leg length	89.50	4.58			84.88	5.82	1.151	8.10	0.81		0.01*
**10MWT/MAT**											
10MWTnormal	1.30 ^b^	1.35 ^c^			2.00 ^b^	1.15 ^c^	-1.078	0.035		-0.597	0.012*^a^
10 MWT fast	1.75 ^b^	0.67 ^c^			1.30 ^b^	0.95 ^c^	-0.339	0.521		0.141	0.518^a^
MAT	9.25 ^b^	2.08 ^c^			14.45 ^b^	2.41 ^c^	-5.923	-3.857		-1.676	0.000*^a^

10MWT = 10Meters Walking Test, MAT = Modified Agility Test, a = Mann-Whitney U, b = Median, c = IQR = Interquartile range, IC = Confidence interval, SD = Stander Deviation

* Significant at p < 0.05

** = p < 0.01

In addition, a breakdown of the IPAQ responses among the female basketball players and non-athletic control group in Saudi Arabia categorized the participants into three activity levels: low, moderate, and high (Table 2). Remarkably, a substantial proportion of the basketball players reported engaging in high physical activity (83.3%), with only a minority reporting moderate activity (16.7%) and none indicating low activity. In stark contrast, most of the non-athletic women reported low physical activity (95.0%), with only a small proportion engaging in moderate activity (5.0%) and none participating in high activity levels. A Fisher Exact test revealed a significant association between activity levels and playing basketball (χ2 = 41.403, p < 0.001), emphasizing the clear disparity in physical activity levels between the two cohorts (Table 2).

**Table 2:** IPAQ analysis.

Group	IPAQ				Fisher Exact test	P-Vale	
	Low	Moderate	High				
Basketball group	0 (0.0%)	3 (16.7%)	15 (83.3%)	41.403			0.000
Non-active group	19 (95.0%)	1 (5.0%)	0 (0.0%)				

IPAQ = International Physical Activity Questionnaire

* significant at = p < 0.01.

The correlation analysis revealed significant negative correlations between IPAQ and 10 MWT normal and MAT, as higher IPAQ scores were associated with better (faster) performance in 10 MWT normal (p < 0.01; r = −0.43, moderate) and MAT (p < 0.01; r = −0.08, large). In addition, we found significant positive correlations between agility (MAT) and 10 MWT normal (p < 0.01; r = 0.55, large). Also, a significant positive correlation was found between 10MWT different speeds (p < 0.01; r = 0.65, large) and as presented in Table 3.

**Table 3:** Correlation between study variables for all subjects (N = 38).

Variable	Spearman’s correlations (r)	IPAQ	10 MWT normal	10 MWT fast	MAT
IPAQ	r	1	_	_	_
	P-value	.	_	_	
10 MWT normal	r	-0.43**	1	_	_
	P-value	0.007	.		_
10MWT fast	r	0.11	0.65**	1	_
	P-value	0.529	0.000	.	_
MAT	r	-0.84**	0.55**	0.10	1
	P-value	0.000	0.000	0.545	.

IPAQ = International Physical Activity Questionnaire, 10MWT = 10Meters Walking Test, MAT = Modified Agility Test

* significant at = p < 0.01.

Furthermore, a subanalysis of a basketball group also showed a positive correlation between 10 MWT’s different speeds (p < 0.01;r = 0.80, large^)^. In addition, positive correlations were found between MAT and 10MWT normal (p < 0.01;r = 0.73, large) and fast (p < 0.01; r = 0.61, large). However, we did not find any significant differences between the years of playing and any other variables (Table 4).

**Table 4:** Correlations between years of playing and all study variables for the basketball group (N = 18).

Variable	Spearman’s correlations (r)	Years of playing	10 MWT normal	10 MWT fast	MAT
Years of playing	r	1	_	_	_
	P-value	.	_	_	_
10MWT normal	r	-0.19	1	_	_
	P-value	0.459	.	.	_
10MWT fast	r	-0.33	0.80**	1	_
	P-value	0.186	0.000	.	_
MAT	r	-0.31	0.73**	0.61**	1
	P-value	0.209	0.001	0.008	.

10MWT = 10Meters Walking Test, MAT = Modified Agility Test, * significant at = p < 0.01.

## 4. Discussion

This study delved into whether there were discernible differences in physical activity levels, speed, and agility values between women basketball players and non-athletic control groups in Saudi Arabia. Our findings, particularly regarding physical performance reveled diffrencess in gait speed and agility which are particularly relevant to basketball performance [15–19]. Our study demostrated that basketball player have faster waking speed and better agility performance compared to compared to the non-athletic control group which are in line with previous research [15–19]. As pervious studies found that playing basketball was associated with superior walking speed and agility performance abilities [15–19]. Previous research showed that basketball players exhibited greater speed than other sports players, such as those in badminton. This finding is not surprising, given that basketball is one of the fastest games in the world, demanding high speed for optimal performance [45, 46]. Furthermore, we found a significant positive correlation between gait speed and agility, which was more robust in the basketball group. These findings align with previous research that reported a significant positive correlation between agility and speed among different sports players [45]. Our participants’ agility scores further support this, indicating that basketball participants perform better in agility tests and spend less time running a distance with direction changes than non-athletic participants. Interstingly, significantly higher agility skills were also reported among basketball players when compared to other sports players, which could be attributed to selected physical fitness components among basketball players [46–48].

Our findings not only confirm the high physical activity levels among the basketball players compared to the non-athletic control group but also shed light on the intricate relationship between their physical activity and performance. Previous studies have shown that engaging in sports significantly increases physical activity levels [15, 19, 38]. Our research further revealed that this physical activity level was negatively correlated with speed and agility times, indicating that a higher level of physical activity was associated with better performance in these areas. These findings align with previous research [9, 14], underscoring their significance and relevance. We also found a negative correlation between years of playing and agility and speed performance, meaning that experienced basketball players have faster speed and better agility skills. However, this correlation was not significant, which could be due to our small sample size of basketball players and not including different levels of basketball players, such as experts versus beginners. Previous studies in senior basketball players have shown significant differences among playing positions for speed and agility [49–53]. Our results and prior studies suggest that playing basketball was associated with higher physical activity, leading to better speed and agility performance [15–53].

Our study focused on female participants as slight differences in physical performance between males and females in various sports have been reported, [54–57] which could be partially explained by biological factors. This may explain, in part, why sports competitions are gender-specific. However, the effects of gender on physical performance are still inconsistent in research, and more studies are needed to confirm gender differences among basketball players. Our study has certain limitations, such as its small sample size, which may affect the generalizability of our results. Also, this study was a cross-sectional study design, which may limit the examination of the within-subject effect of playing basketball among our participants. Although this study aims to compare physical activity and performance between basketball players and non-athletic control groups and does not necessarily measure the effect of playing basketball, future studies should focus on engaging, motivating, and increasing the activity levels of those in the less active group. Raising awareness and promoting women’s basketball participation should be recommended, which could be used as a way to enhance their overall physical activity levels and speed and agility capabilities.

## 5. Conclusions

We conclude that the individuals who regularly played basketball exhibited superior physical activity levels and higher physical performance in speed and agility compared to those who were less active. Physical activity levels and years of playing basketball were associated with better physical performance capabilities. Nevertheless, these findings could be used in future prospective studies to evaluate the physical performance of basketball players. Furthermore, a more extensive study is needed to establish normative values regarding physical characteristics and fitness performance measurement (i.e. heights, BMI, 10MWT, MAT, and IPAQ) among basketball players in Saudi Arabia using a larger sample size. We included only female participants; further studies are required to compare physical performance among female and male basketball players in Saudi Arabia. Further study of anthropometric and performance measures and their relation to each other is necessary for overall performance enhancement in the basketball community. Additional research is also needed to investigate the effects of years of basketball playing on different physical performances among different levels of players, such as experienced and beginner basketball players in Saudi Arabia.

## Supporting information

S1 Checklist STROBE checklist. (DOC)
